# Cyberchondria, Psychological Well-Being, Anxiety, and Depression Levels in Female Patients with Irritable Bowel Syndrome

**DOI:** 10.3390/medicina62091621

**Published:** 2026-08-22

**Authors:** Esra Suay Timurkaan, Mustafa Timurkaan, Sevler Yıldız

**Affiliations:** 1 Department of Internal Medicine, Fethi Sekin City Hospital, Elazig 23280, Turkey; dresrasuay@gmail.com; 2 Department of Psychiatry, Fethi Sekin City Hospital, University of Health Sciences, Elazig 23280, Turkey; dr_sevler@hotmail.com

**Keywords:** irritable bowel syndrome, cyberchondria, psychological well-being, anxiety, depression

## Abstract

*Background and Objectives*: This study evaluated the interplay between cyberchondria, psychological distress, and symptom severity in female patients with irritable bowel syndrome (IBS). *Materials and Methods*: The study included 176 female participants: 86 patients with IBS and 90 controls. Participants completed a sociodemographic form, the IBS Symptom Severity Score (IBS-SSS), Cyberchondria Severity Scale–Short Form (CSS-12), Psychological Well-Being Scale (PWBS), Beck Anxiety Inventory (BAI), and Beck Depression Inventory (BDI). *Results*: Compared with healthy controls, women with IBS had significantly higher scores on the BAI (*p* < 0.001), BDI (*p* < 0.001), and CSS-12 Distress (*p* = 0.003), Reassurance (*p* < 0.001), and Compulsion (*p* < 0.001) subscales, whereas CSS-12 Excessiveness did not differ significantly between the groups (*p* = 0.211). Unexpectedly, PWBS scores were also significantly higher in women with IBS than in healthy controls (*p* < 0.001). IBS-SSS scores were significantly higher in the IBS group (*p* < 0.001). Within the IBS group, IBS-SSS was positively correlated with BAI (r = 0.255, *p* = 0.018) but not with BDI (r = 0.099, *p* = 0.365), while BDI was negatively correlated with PWBS (r = −0.320, *p* = 0.003). PWBS showed a weak positive correlation with CSS-12 Excessiveness (r = 0.215, *p* = 0.046), but not with the other CSS-12 dimensions. In ROC analysis, BAI (cut-off > 3) showed 88.4% sensitivity and 82.2% specificity; BDI (cut-off > 4), 82.6% and 86.7%; PWBS (cut-off > 33), 70.9% and 98.9%; and IBS-SSS (cut-off > 50), 96.5% and 88.9%, respectively. *Conclusions*: It was found that women with IBS had higher anxiety and depressive symptom scores compared with healthy female, and that there were differences in various dimensions of cyberchondria. The unexpectedly higher psychological well-being scores observed in the IBS group should be interpreted with caution. These findings point to the potential importance of psychological evaluation as part of a comprehensive assessment of IBS. Effect sizes for the between-group differences were large for the BAI and BDI (Cliff’s delta = 0.74 for both), the CSS-12 Reassurance subscale (delta = 0.53), the PWBS (Cohen’s d = 1.79) and the IBS-SSS (d = 2.84), and small for the Distress (delta = 0.28) and Compulsion (delta = 0.27) subscales.

## 1. Introduction

Irritable bowel syndrome (IBS) is a chronic and recurrent gastrointestinal disorder characterized by symptoms such as bloating, abdominal pain, and changes in bowel habits, which can significantly affect the quality of life of individuals with this condition [1]. IBS is currently conceptualized as a condition involving gut–brain interactions and is understood within a biopsychosocial framework, involving not only physiological processes but also psychological factors that may substantially affect patients’ quality of life [2]. A 2025 meta-analysis covering 52 countries reported that the prevalence of this increasingly common disorder worldwide is 14.1% [3]. The prevalence of IBS in women is reported to be higher than in men, with a female-to-male ratio of roughly 2:1 [4].

Psychiatric comorbidities are prevalent among individuals with IBS. In particular, anxiety and depression are more prevalent among individuals with IBS than in the general population [5]. A meta-analysis reported that the combined prevalence of anxiety symptoms and disorders in patients with irritable bowel syndrome (IBS) was 39.1%, while the prevalence of depressive symptoms and disorders was estimated to be 28.8% [6]. The effects of psychosocial stressors on the gastrointestinal system are explained through the “gut–brain axis.” Psychosocial stress may exacerbate intestinal symptoms by affecting intestinal motility, mucosal permeability, and visceral sensitivity [7]. At the neurotransmitter level, serotonin (5-HT), dopamine, gamma-aminobutyric acid (GABA), and norepinephrine play important roles in the pathophysiology of IBS. Serotonin is one of the most abundant neurotransmitters in the gastrointestinal system and plays an important role in intestinal motility, secretion, and visceral sensitivity. Serotonin imbalances have been shown to be associated with different clinical symptoms in diarrhea-predominant subtypes as well as constipation-predominant IBS [8,9]. Furthermore, it is thought that other neurotransmitters, such as GABA and norepinephrine, may influence gastrointestinal symptoms via the gut–brain axis by modulating anxiety and stress responses [10]. Based on an evaluation of these findings, it is thought that changes in neurotransmitter systems may play a role in the complex interaction between gastrointestinal symptoms and psychiatric comorbidities.

The concept of psychological well-being refers to an individual’s self-actualization, establishing positive relationships with their environment, and their capacity to enjoy life [11]. According to Ryff’s (1989) model, this concept, which refers to the maintenance of an individual’s optimal level of functioning, consists of sub-dimensions such as autonomy, self-acceptance, purpose in life, environmental mastery, personal growth, and positive relationships [12]. It has been shown that psychological well-being is associated not only with psychological factors such as cognitive flexibility and self-efficacy but also with neurobiological and stress-related processes [13]. Research indicates that psychological well-being in IBS patients is significantly lower than in healthy controls [14]. It has been demonstrated that subjective well-being levels in diseases affecting the gastrointestinal system are inversely related to disease severity, anxiety, and depression [15]. Cyberchondria, an emerging psychosocial aspect of the digital age, may also be associated with lower psychological well-being in patients with IBS. Cyberchondria is characterized by individuals repeatedly searching for health information online in order to reduce their health-related concerns [16]. The chronic and variable nature of IBS may encourage individuals with this condition to seek health-related information online. Exposure to inaccurate health information obtained through online information seeking may be associated with increased health anxiety [17]. The activation of stress-related pathways may be associated with physiological stress responses involving the hypothalamic–pituitary–adrenal axis, as well as decreased cognitive flexibility and changes in perceived self-efficacy [18]. Research shows that women engage in health-related information-seeking behavior on the internet more frequently than men [19]. The fact that women generally have higher levels of health anxiety and show greater sensitivity to health-related information may explain the gender differences in cyberchondriac behavior [20]. Consequently, these observations suggest that cyberchondria may be a relevant psychosocial factor to investigate in women with IBS, particularly in relation to psychological symptoms and well-being. The objective of this study was to compare cyberchondria-related behaviors, anxiety and depressive symptoms, and psychological well-being between women with IBS and controls, and to examine their associations with IBS symptom severity.

## 2. Materials and Methods

This study was approved by the Elazig Fethi Sekin City Hospital Non-Interventional Clinical Research Ethics Committee (Decision No: 2025/3-17, Date: 6 February 2025), and was conducted in accordance with the ethical principles outlined in the Declaration of Helsinki (2013 revision). The study included women aged 18–65 years who were clinically diagnosed with IBS according to the Rome IV criteria. All participants were fully informed about the aims and procedures of the study and provided written informed consent prior to enrolment. Individuals who were unable to provide informed consent were not included in the study, and no minors or other legally vulnerable populations were enrolled. The present study employed a cross-sectional observational design. Female patients (n = 100) who were diagnosed with irritable bowel syndrome (IBS) and who visited the Internal Medicine Outpatient Clinic at Fethi Hospital between March 2025 and June 2025 were included in the study. After being evaluated by an internal medicine specialist, they were referred to a psychiatrist. After the patients’ mental health examinations, the duration of the disease, treatment history, and other sociodemographic data were recorded. Restricting the study population to women reduced between-sex variability; however, reproductive stage, menopausal status, hormonal factors, and gender-related social circumstances were not specifically assessed. The Rome IV criteria were used for the diagnosis of IBS. According to the Rome IV criteria, IBS is defined by recurrent abdominal pain occurring, on average, at least 1 day per week during the previous 3 months, with symptom onset at least 6 months before diagnosis. In addition, abdominal pain must be accompanied by at least two of the following criteria for diagnosis: (a) related to defecation, (b) change in defecation frequency, (c) change in stool form. No new treatment that could affect bowel function was started during participation in the study, and no changes were made to existing treatments in the last month. Women with organic gastrointestinal diseases, psychiatric disorders, and systemic diseases were excluded from this study. The presence of psychiatric diseases among participants was assessed through clinical psychiatric interviews and mental status examinations conducted by a psychiatrist, as well as through a review of existing medical documentation in the hospital’s record system. Participants in whom an existing psychiatric disorder was identified as a result of these assessments were excluded from the study. Additionally, a control group was formed from healthy volunteers (n = 100) who had come to our hospital for routine annual check-ups, had no known psychiatric or systemic diseases, and had no active complaints. All individuals signed an informed consent form, and all participants completed a sociodemographic data form, Irritable Bowel Syndrome Symptom Severity Score (IBS-SSS), Cyberchondria Severity Scale–Short Form (CSS-12), Psychological Well-Being Scale, Beck Anxiety Inventory (BAI), and Beck Depression Inventory (BDI). Nine patients diagnosed with IBS voluntarily withdrew from the study, while five patients were excluded from the study for failing to complete the scales. Similarly, three individuals from the healthy control group withdrew from the study, while seven were excluded from the study for failing to complete the scales.

### 2.1. Scales Used in the Study

*Sociodemographic form:* This form was prepared by the researchers based on a review of the literature relevant to the purpose and scope of the study. It was designed to collect demographic and clinical data (such as age, gender, educational status, place of residence, and family characteristics) from the individuals included in the study.

*Beck Depression Inventory (BDI):* This scale was developed to assess the severity of depressive symptoms in adults. The total score obtained from the scale is evaluated as 0–9 no/minimal depression, 10–18 mild depression, 19–29 moderate depression, 30–63 severe depression. The validity and reliability of the Turkish version of this scale have been previously established [21,22].

*Beck Anxiety Inventory (BAI):* Developed by Beck and colleagues, the BAI is a self-report scale used to assess the severity of anxiety symptoms. A total score of 8–15 indicates low anxiety, 16–25 indicates moderate anxiety, and 26–63 indicates high anxiety. The validity and reliability of the Turkish version of this scale have been previously established [23,24].

*Cyberchondria Severity Scale–Short Form (CSS-12):* The 12-item short form of the scale originally consisting of 43 items, developed by McElroy and Shevlin, was created by McElroy and colleagues [24]. The validity and reliability of the Turkish version have been established. The scale is a five-point Likert scale, and the score ranges from 0 to 60. A high score indicates a high level of cyberchondria. It has subscales for excessiveness, distress, reassurance, and compulsion. Excessiveness is thought to represent the increasing and repetitive nature of the searches, distress represents increased anxiety/distress levels as a result of the searches, the reassurance subscale represents searches that lead individuals to seek professional medical advice, and the compulsion subscale represents web searches that affect all aspects of life [25,26].

*Psychological Well-Being Scale:* This eight-item scale developed by Diener and colleagues (2009) covers various dimensions of human functioning, ranging from positive relationships to a sense of competence and a meaningful and purposeful life. The items of the Psychological Well-Being Scale are rated on a 7-point Likert-type scale ranging from “strongly disagree” (1) to “strongly agree” (7). All items are phrased in a positive manner. The total score that can be obtained from the scale ranges from 8 to 56. Higher scores indicate greater psychological well-being. The Turkish validity and reliability study of the scale was conducted by Telef [27,28].

*Irritable Bowel Syndrome Symptom Severity Score (IBS-SSS):* This scale, which contains questions for individuals complaining of abdominal pain based on the nature and duration of their pain, has a total score range of 0–500. The total score is categorized as follows: below 75, 75–174 (mild), 175–299 (moderate), and 300 and above (severe) [29].

The Beck Depression Inventory, Beck Anxiety Inventory, and Psychological Well-Being Scale were administered using their previously published and validated Turkish versions. The IBS Symptom Severity Score (IBS-SSS) was administered in Turkish; however, a formally published Turkish validation study of this scale was not available. No scale was specifically translated for the purposes of the present study.

### 2.2. Statistical Analysis

In this study, analyses were performed using the SPSS 23.0 (Statistical Package for the Social Sciences; SPSS Inc., Chicago, IL, USA) 22 software packages. Descriptive data were presented as n and % values for categorical variables and as mean ± standard deviation (Mean ± SD) and median (25th–75th percentile range) for continuous variables. Chi-square analysis (Pearson Chi-square test) was applied to compare categorical variables between groups. The normality of continuous variables was assessed using the Kolmogorov–Smirnov test. For variables showing a normal distribution, the difference between two groups was analyzed using the Student *t*-test, while for variables not showing a normal distribution, the Mann–Whitney U-test was used. Because at least one variable in every pair deviated from a normal distribution in the Kolmogorov–Smirnov test, relationships between continuous variables were examined using Spearman’s rank-order correlation analysis; no Pearson correlation coefficients are reported. ROC (Receiver Operating Characteristic) curve analyses were performed to evaluate the ability of the scale scores to discriminate between participants with and without IBS. A significance level of *p* < 0.05 was accepted in all statistical analyses. All statistical tests were two-tailed. Effect sizes were computed for all between-group comparisons: Cohen’s d with 95% confidence intervals (CI) for variables compared with Student’s *t*-test, and Cliff’s delta with 95% CI for variables compared with the Mann–Whitney U test. Cohen’s d values of 0.20, 0.50 and 0.80 and Cliff’s delta values of 0.15, 0.33 and 0.47 were interpreted as small, medium and large effects, respectively.

## 3. Results

A total of 176 female participants were included in this study; 86 of these patients had irritable bowel syndrome, while 90 were in the healthy control group. No significant differences were observed between groups in terms of age (*p* = 0.960), marital status (*p* = 0.677), and educational status (*p* = 0.056). The employment rate among patients (75.6%) was significantly higher than that among controls (45.6%) (*p* < 0.001). The median duration of diagnosis among patients was 1.0 (0.5–3.0) years. (Table 1).

In the patient group, scores for the BAI (*p* < 0.001), BDI (*p* < 0.001), CSS-12 Distress subscale (*p* = 0.003), CSS-12 reassurance subscale (*p* < 0.001), CSS-12 compulsion subscale (*p* < 0.001), PWBS (*p* < 0.001), and IBS Symptom Severity Scale (*p* < 0.001) were found to be significantly higher than those of the control group (Table 2). The between-group effect sizes were large for the BAI (Cliff’s delta = 0.74, 95% CI 0.62 to 0.86), the BDI (delta = 0.74, 95% CI 0.63 to 0.86) and the CSS-12 Reassurance subscale (delta = 0.53, 95% CI 0.40 to 0.67), and small for the CSS-12 Distress (delta = 0.28, 95% CI 0.12 to 0.45) and Compulsion (delta = 0.27, 95% CI 0.11 to 0.43) subscales, whereas the effect size for CSS-12 Excessiveness was negligible (delta = −0.09, 95% CI −0.26 to 0.08). For the variables compared with Student’s *t*-test, large effects were observed for both the PWBS (Cohen’s d = 1.79, 95% CI 1.44 to 2.14) and the IBS-SSS (d = 2.84, 95% CI 2.42 to 3.26), whereas age did not differ between the groups (d = −0.01, 95% CI −0.30 to 0.29) (Table 2).

When the scores were classified according to the severity categories defined for each instrument, 50.0% (n = 43) of the women with IBS scored in the minimal range of the BDI, whereas 27.9% (n = 24) scored in the mild, 12.8% (n = 11) in the moderate and 9.3% (n = 8) in the severe range; the corresponding proportions in the control group were 93.3% (n = 84), 2.2% (n = 2), 1.1% (n = 1) and 3.3% (n = 3) (*p* < 0.001). On the BAI, 26.7% (n = 23) of the patients scored below the threshold for low anxiety, while 30.2% (n = 26) were in the low, 32.6% (n = 28) in the moderate and 10.5% (n = 9) in the high anxiety range, compared with 90.0% (n = 81), 4.4% (n = 4), 2.2% (n = 2) and 3.3% (n = 3) of the controls, respectively (*p* < 0.001). Overall, at least mild depressive symptoms were present in 50.0% of the patients versus 6.7% of the controls, and at least low-level anxiety symptoms in 73.3% versus 10.0%, respectively. According to the IBS-SSS categories, 7.0% (n = 6) of the patients scored below 75, while 27.9% (n = 24) had mild, 58.1% (n = 50) moderate and 7.0% (n = 6) severe symptoms.

**Figure 1:**
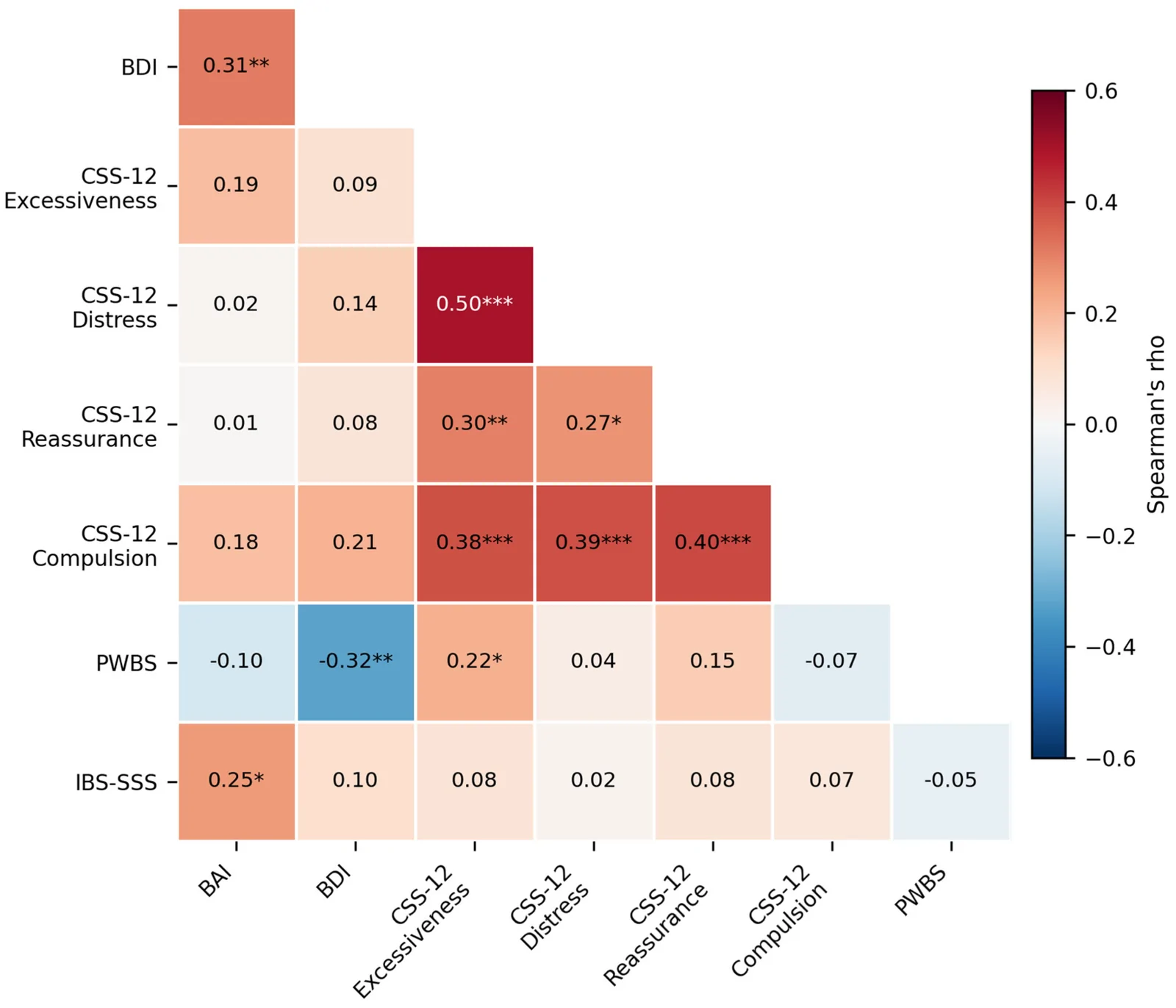
Spearman correlation matrix of the scale scores in the IBS patient group (n = 86). Cell values are Spearman’s rho. * *p* < 0.05; ** *p* < 0.01; *** *p* < 0.001. BAI, Beck Anxiety Inventory; BDI, Beck Depression Inventory; CSS-12, Cyberchondria Severity Scale–Short Form; PWBS, Psychological Well-Being Scale; IBS-SSS, Irritable Bowel Syndrome Symptom Severity Score.

Only BAI, but not BDI, was correlated with IBS symptom severity in the irritable bowel syndrome patient group. In the IBS group, IBS symptom severity was significantly, although weakly, positively correlated with anxiety symptoms (r = 0.255, *p* = 0.018), whereas no significant correlation was observed between IBS symptom severity and depressive symptoms (r = 0.099, *p* = 0.365). Depressive symptoms were negatively correlated with psychological well-being (r = −0.320, *p* = 0.003). Among the CSS-12 subscales, Excessiveness was positively correlated with Distress (r = 0.495, *p* < 0.001), Reassurance (r = 0.302, *p* = 0.005), Compulsion (r = 0.380, *p* < 0.001), and PWBS (r = 0.215, *p* = 0.046). Distress was positively correlated with Reassurance (r = 0.270, *p* = 0.012) and Compulsion (r = 0.387, *p* < 0.001), while Reassurance was positively correlated with Compulsion (r = 0.398, *p* < 0.001) (Table 3). All coefficients were obtained with Spearman’s rank-order correlation; the correlation matrix of the patient group is also presented graphically in Figure 1.

**Figure 2:**
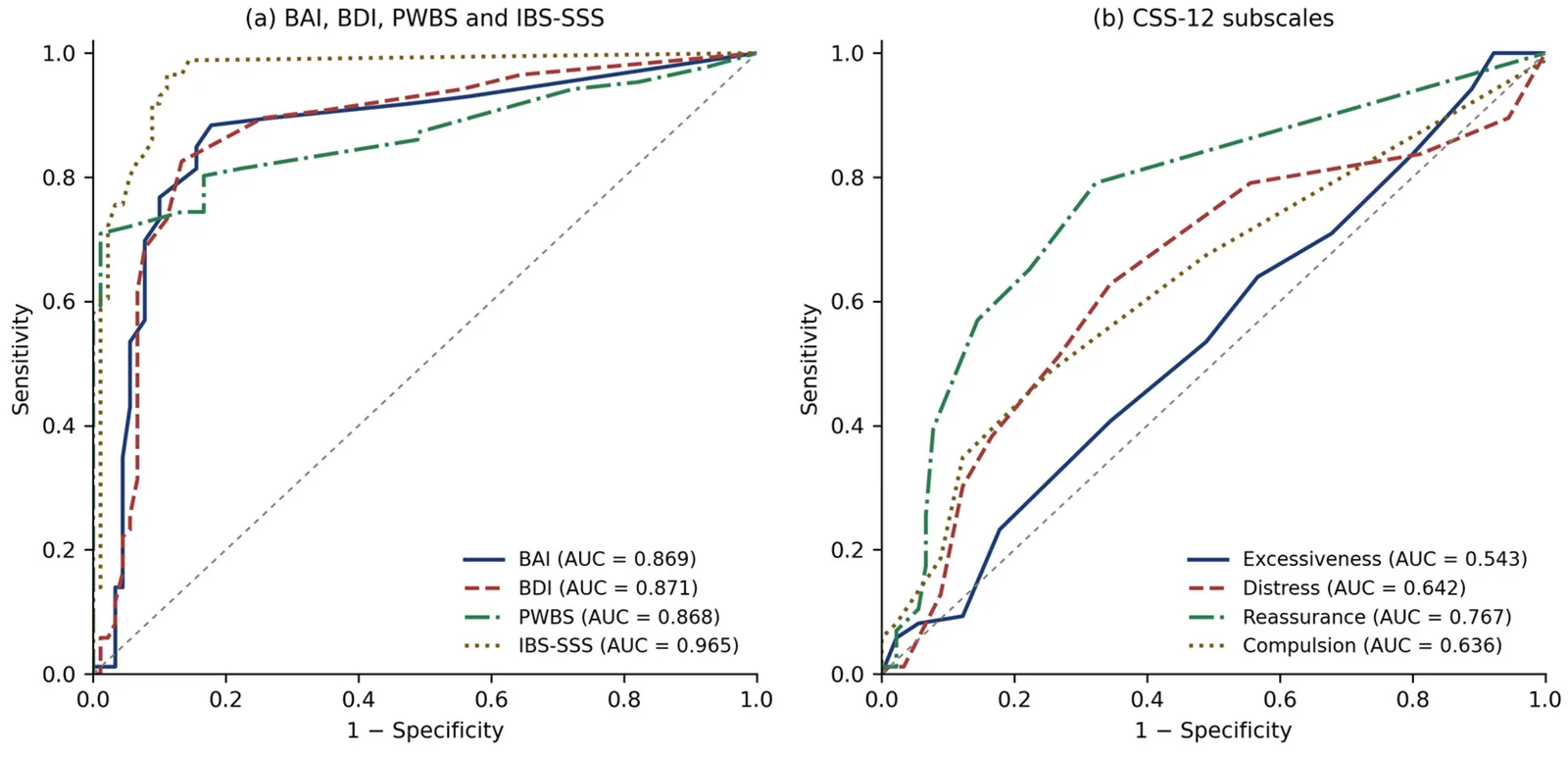
Receiver operating characteristic (ROC) curves of the scale scores for discriminating between participants with and without IBS: (**a**) BAI, BDI, PWBS and IBS-SSS; (**b**) CSS-12 subscales. For the CSS-12 Excessiveness subscale the curve corresponds to the direction reported in Table 4 (cut-off ≤ 13). The diagonal line represents no discrimination (AUC = 0.500). AUC, area under the curve.

The ability of the scale scores to discriminate between participants with and without IBS was evaluated using ROC curve analysis. When a cut-off value of 3 was used for the BAI, a sensitivity of 88.4% and a specificity of 82.2% were found. When a value of 4 was taken as the cut-off for the BDI, a sensitivity of 82.6% and a specificity of 86.7% were determined. When a value of 13 was taken as the cut-off for the CSS-12-excessiveness subscale, a sensitivity of 100% and a specificity of 7.8% were determined. When a cutoff value of 6 was used for the CSS-12 distress sub-scale, a sensitivity of 62.8% and a specificity of 65.6% were determined. When a cutoff value of 3 was used for the CSS-12 Reassurance subscale, a sensitivity of 79.1% and a specificity of 67.8% were determined. When a cut-off value of 4 was used for the CSS-12 Compulsion subscale, a sensitivity of 48.8% and a specificity of 74.4% were determined. When a cutoff value of 33 was used for PWBS, a sensitivity of 70.9% and a specificity of 98.9% were determined. When a value of 50 was used as the cut-off point for the IBS Symptom Severity Score Questionnaire, a sensitivity of 96.5% and a specificity of 88.9% were determined (Table 4). The corresponding ROC curves are presented in Figure 2.

## 4. Discussion

In the current study, we aimed to evaluate the associations between cyberchondria tendencies and psychological symptoms in women diagnosed with IBS. Furthermore, women with IBS had significantly higher scores on the Distress, Reassurance, and Compulsion dimensions of cyberchondria than healthy controls, whereas no significant between-group difference was observed in Excessiveness. In addition, ROC curve analysis showed varying levels of discrimination between participants with and without IBS for the IBS-SSS, BAI, BDI, and PWBS.

Previous research has demonstrated that IBS is closely associated with anxiety and depression [30]. Compared to the healthy group, IBS patients were three times more likely to experience symptoms of anxiety or depression [6]. It has even been reported that psychological symptoms can alter visceral perception and affect the microbiome–gut–brain axis, thereby exacerbating gastrointestinal symptoms [31]. Considering the higher prevalence of IBS in women, it has been reported that women with IBS are more likely to experience a higher burden of psychological comorbidities, including anxiety and depression [4]. The reasons for these gender-based differences include changes in estrogen and progesterone levels, differences in the composition of the gut microbiota, visceral sensitivity, and differences in immune function [32]. Some studies have suggested that relatively high stress levels in women may exacerbate IBS symptoms through neuroendocrine and immune pathways [33]. In our study, the median BAI and BDI scores in women with IBS were 14.5 (7.0–18.0) and 9.5 (5.0–15.0), respectively. This finding is partly consistent with the literature suggesting that IBS is often associated with psychological factors, but it differs from the high rates of anxiety and depression reported in some studies. This suggests that psychological symptoms in individuals with IBS are heterogeneous and that not all individuals with IBS have a clear psychiatric comorbidity. It also supports the notion that IBS is a complex disorder associated not only with psychological factors but also with neurogastroenterological and immunological mechanisms. Therefore, the current findings characterize the profile of psychological symptoms observed in women with IBS; however, comparative studies with larger samples that include both women and men are needed to determine whether there are sex-related differences in the psychological variables of IBS. An unexpected finding of the present study was that psychological well-being scores were significantly higher in women with IBS than in healthy controls (36.7 ± 10.9 vs. 21.3 ± 5.7, *p* < 0.001). Given that higher scores on the PWBS indicate greater psychological resources, this finding was contrary to the anticipated direction and should be interpreted cautiously. Notably, within the IBS group, depressive symptoms were negatively correlated with psychological well-being (r = −0.320, *p* = 0.003), indicating that higher depressive symptom levels were associated with lower psychological well-being despite the higher overall well-being scores observed in the IBS group. The between-group difference in psychological well-being may have been influenced by unmeasured factors and by sociodemographic differences between the groups. In particular, the significant variation in employment status between the IBS and control groups should be considered when interpreting this finding.

Cyberchondria is linked to increased anxiety, an increased frequency of online health information searches, and changes in medical care-seeking behaviors; it may be especially relevant in individuals with conditions involving complex interactions between psychological and physical factors [34,35]. It has been reported that cyberchondria is associated with various psychological factors, including obsessive–compulsive symptoms, intolerance of uncertainty, and negative cognitive evaluation of health-related thoughts [36]. A study of 431 individuals examining the effect of health anxiety on the relationship between somatic symptoms and cyberchondria reported that the severity of somatic symptoms predicted increasing levels of cyberchondria and that health anxiety partially influenced this relationship [37]. Our findings showed higher Distress, Reassurance, and Compulsion scores in women with IBS than in healthy controls, whereas Excessiveness scores did not differ significantly between the groups. The search for internet-based information among IBS patients may stem from the uncertainty of symptoms as well as the need for control arising from the chronic nature of the disease [38]. While this may sometimes facilitate the search for professional medical help, it may also contribute to increased anxiety through exposure to inaccurate or exaggerated health information [39]. IBS is a complex disorder intertwined with psychological and cognitive processes in addition to gastrointestinal symptoms [40]. Poorer psychological well-being may be associated with both the experience of the disease and online health information-seeking behaviors [41]. It has been noted that even low levels of psychological distress can play a significant role in symptom perception and coping strategies [42,43]. In this study, while a weak positive correlation was found between psychological well-being and the “Excessiveness” subscale of cyberchondria, no statistically significant correlation was found between psychological well-being and the other CSS-12 subscales. These results indicate that the relationships between psychological well-being and the various dimensions of cyberchondria may reflect a heterogeneous pattern and that these relationships should be interpreted with caution.

The relatively low BAI (>3) and BDI (>4) cut-off values identified in the ROC analyses should not be interpreted as clinical thresholds for anxiety or depression. Rather, these data-driven cut-off values reflect the discrimination between the selected IBS and healthy control groups in the current sample and may partly reflect the very low symptom scores observed in the control. Notably, the CSS-12 Excessiveness subscale did not differ significantly between the IBS and control groups (*p* = 0.211) and showed limited discriminatory performance in the ROC analysis (AUC = 0.543, *p* = 0.315), with a specificity of only 7.8%. Therefore, the Excessiveness dimension should not be considered useful for differentiating between participants with and without IBS in the present sample.

Our study has several limitations. The study has a cross-sectional design; therefore, causal inferences cannot be drawn from the observed associations. Given the cross-sectional design, the directionality of the associations between cyberchondria-related digital health behaviors and IBS symptom severity cannot be established. Accordingly, longitudinal studies are needed. Although restricting the study population to women reduced between-sex variability, reproductive stage, menopausal status, hormonal factors, and gender-related social factors were not assessed and may have influenced the observed associations. The data were obtained through self-report questionnaires; therefore, the findings may be subject to response bias, particularly for subjective measures. Measurement invariance between the IBS and control groups was not assessed; therefore, between-group comparisons should be interpreted with caution, as the equivalence of the measurement properties of the instruments across the two groups was not established. Future longitudinal studies incorporating relevant biological and neurophysiological measures are needed to further clarify the relationships observed in the current study.

## 5. Conclusions

While women with IBS were found to have higher levels of anxiety, depressive symptoms, and certain dimensions of cyberchondria compared to controls, their psychological well-being scores were unexpectedly higher. Although these findings should be interpreted with caution, they suggest that psychological assessment may provide complementary information in the comprehensive evaluation of individuals with IBS. However, these scales should not be interpreted as diagnostic or predictive tools for IBS. These findings suggest that focusing solely on symptom scores in clinical practice may be insufficient and that psychological evaluations may provide complementary information. Psychological well-being levels may offer valuable information in terms of both symptom management and patient quality of life. In this context, addressing both the physical and psychological components of IBS may contribute to a more comprehensive approach to treatment and follow-up.

## Figures and Tables

**Table 1:** Demographic comparison of the groups.

		Patient		Control		*p*
		n	%	n	%	
Age, Mean ± SD		37.8 ± 7.7		37.9 ± 11.8		0.960 *
Marital status	Married	70	81.4	71	78.9	0.677 **
	Single	16	18.6	19	21.1	
Educational background	High school or below	28	32.6	42	46.7	0.056 **
	University/College	58	67.4	48	53.3	
Employment status	Employed	65	75.6	41	45.6	<0.001 **
	Unemployed	21	24.4	49	54.4	
Diagnosis period, Median (IQR)		1.0 (0.5–3.0)		-		-
Smoking	Yes	17	19.8	17	18.9	0.883 **
	No	69	80.2	73	81.1	

* Student’s *t*-test and ** chi-square analysis were applied.

**Table 2:** Comparison of all characteristics of the groups.

	Patient	Control	*p*	Effect Size (95% CI)
	Median (IQR)	Median (IQR)		
Beck Anxiety Inventory	14.5 (7.0–18.0)	1.0 (0.0–3.0)	<0.001 *	0.74 (0.62 to 0.86)
Beck Depression Inventory	9.5 (5.0–15.0)	2.0 (0.0–4.0)	<0.001 *	0.74 (0.63 to 0.86)
CSS-12: Cyberchondria Severity Scale–Short Form-Excessiveness	8.0 (7.0–11.0)	9.0 (7.0–11.0)	0.211 *	−0.09 (−0.26 to 0.08)
CSS-12: Cyberchondria Severity Scale–Short Form-Distress	8.0 (6.0–10.0)	6.0 (5.0–8.0)	0.003 *	0.28 (0.12 to 0.45)
CSS-12: Cyberchondria Severity Scale–Short Form-Reassurance	6.0 (4.0–8.0)	3.0 (3.0–4.0)	<0.001 *	0.53 (0.40 to 0.67)
CSS-12: Cyberchondria Severity Scale–Short Form-Compulsion	4.0 (3.0–6.0)	3.0 (3.0–5.0)	<0.001 *	0.27 (0.11 to 0.43)
Psychological well-being scale, Mean ± SD	36.7 ± 10.9	21.3 ± 5.7	<0.001 **	1.79 (1.44 to 2.14)
Irritable Bowel Syndrome Symptom Severity Score, Mean ± SD	197.3 ± 75.1	17.7 ± 49.4	<0.001 **	2.84 (2.42 to 3.26)

* The Mann–Whitney U test and ** Student’s *t*-test were applied. All tests were two-tailed. Effect size: Cliff’s delta for variables compared with the Mann–Whitney U test and Cohen’s d for variables compared with Student’s *t*-test.

**Table 3:** Correlation of scale scores in the patient group.

		Beck Anxiety Inventory	Beck Depression Inventory	CSS-12: Cyberchondria Severity Scale–Short Form -Excessiveness	CSS-12: Cyberchondria Severity Scale–Short Form -Distress	CSS-12: Cyberchondria Severity Scale–Short Form -Reassurance	CSS-12: Cyberchondria Severity Scale–Short Form -Compulsion	Psychological Well-Being Scale, Mean ± SD	Irritable Bowel Syndrome Symptom Severity Score, Mean ± SD
Beck Depression Inventory	r	0.314							
	*p*	0.003							
CSS-12: Cyberchondria Severity Scale–Short Form -Excessiveness	r	0.186	0.092						
	*p*	0.086	0.400						
CSS-12: Cyberchondria Severity Scale–Short Form -Distress	r	0.016	0.141	0.495					
	*p*	0.885	0.196	<0.001					
CSS-12: Cyberchondria Severity Scale–Short Form -Reassurance	r	0.007	0.079	0.302	0.270				
	*p*	0.947	0.470	0.005	0.012				
CSS-12: Cyberchondria Severity Scale–Short Form -Compulsion	r	0.180	0.207	0.380	0.387	0.398			
	*p*	0.097	0.056	<0.001	<0.001	<0.001			
Psychological well-being scale, Mean ± SD	r	−0.104	−0.320	0.215	0.043	0.148	−0.067		
	*p*	0.340	0.003	0.046	0.698	0.174	0.543		
Irritable Bowel Syndrome Symptom Severity Score, Mean ± SD	r	0.255	0.099	0.082	0.022	0.079	0.066	−0.050	
	*p*	0.018	0.365	0.454	0.837	0.470	0.547	0.644	
Age	r	−0.042	−0.014	0.031	0.107	−0.036	−0.134	−0.052	−0.057
	*p*	0.700	0.900	0.778	0.327	0.745	0.218	0.637	0.602
Diagnosis period	r	0.008	0.172	0.032	0.253	0.176	0.015	−0.154	−0.061
	*p*	0.955	0.232	0.824	0.076	0.220	0.918	0.287	0.673

All correlation coefficients were calculated using Spearman’s rank-order correlation (rho); values in bold indicate *p* < 0.05. Correlations were computed within the IBS patient group only (n = 86).

**Table 4:** ROC Analysis of Scale Scores for Discriminating Between Participants With and Without IBS.

Scale and Cut-Off Value	AUC	*p*	95 Percent Confidence İnterval		Sensitivity (%)	Specificity (%)	PPV (Positive Predictive Value) (%)	NPV (Negative Predictive Value) (%)
			Lower Limit	Upper Limit				
Beck Anxiety Inventory > 3	0.869	<0.001	0.810	0.915	88.4	82.2	82.6	88.1
Beck Depression Inventory > 4	0.871	<0.001	0.813	0.917	82.6	86.7	85.5	83.9
CSS-12: Cyberchondria Severity Scale–Short Form -Excessiveness ≤ 13	0.543	0.315	0.467	0.619	100	7.8	50.9	100
CSS-12: Cyberchondria Severity Scale–Short Form -Distress > 6	0.642	0.001	0.566	0.712	62.8	65.6	63.5	64.8
CSS-12: Cyberchondria Severity Scale–Short Form -Reassurance > 3	0.767	<0.001	0.697	0.827	79.1	67.8	70.1	77.2
CSS-12: Cyberchondria Severity Scale–Short Form -Compulsion > 4	0.636	0.001	0.560	0.707	48.8	74.4	64.6	60.4
Psychological well-being scale, Mean ± SD > 33	0.868	<0.001	0.809	0.914	70.9	98.9	98.4	78.1
Irritable Bowel Syndrome Symptom Severity Score > 50	0.965	<0.001	0.926	0.987	96.5	88.9	89.2	96.4

## Data Availability

De-identified participant data and analysis are provided. Access to any potentially identifiable information is restricted by Elazig Fethi Sekin City Hospital Ethics Board and will be granted upon reasonable request to [contact: mustafatimurkan@gmail.com], subject to institutional approvals.
